# Patterns of distant metastasis and survival outcomes in *de novo* metastatic breast cancer according to age groups

**DOI:** 10.3389/fendo.2024.1385756

**Published:** 2024-05-01

**Authors:** Ke Liu, An-Le Huang, Xue-Qin Chen, San-Gang Wu

**Affiliations:** ^1^ Xiamen Key Laboratory of Clinical Efficacy and Evidence Studies of Traditional Chinese Medicine, The First Affiliated Hospital of Xiamen University, School of Medicine, Xiamen University, Xiamen, China; ^2^ Department of Gastrointestinal Oncology Surgery, The First Affiliated Hospital of Xiamen University, School of Medicine, Xiamen University, Xiamen, China; ^3^ Department of Radiation Oncology, Xiamen Cancer Quality Control Center, Xiamen Cancer Center, Xiamen Key Laboratory of Radiation Oncology, The First Affiliated Hospital of Xiamen University, School of Medicine, Xiamen University, Xiamen, China

**Keywords:** breast cancer, age, prognosis, metastatic patterns, SEER

## Abstract

**Background:**

Is *de novo* metastatic breast cancer (dnMBC) the same disease in the elderly as in younger breast cancer remains unclear. This study aimed to determine the metastatic patterns and survival outcomes in dnMBC according to age groups.

**Methods:**

We included patients from the Surveillance Epidemiology and End Results program. Chi-square test, multivariate logistic regression analyses, and multivariate Cox regression models were used for statistical analyses.

**Results:**

A total of 17719 patients were included. There were 3.6% (n=638), 18.6% (n=3290), 38.0% (n=6725), and 39.9% (n=7066) of patients aged <35, 35-49, 50-64, and ≥65 years, respectively. Older patients had a significantly higher risk of lung metastasis and a significantly lower risk of liver metastasis. There were 19.1%, 25.6%, 30.9%, and 35.7% of patients with lung metastasis in those aged <35, 35-49, 50-64, and ≥65 years, respectively. Moreover, the proportion of liver metastasis was 37.6%, 29.5%, 26.3%, and 19.2%, respectively. Age was the independent prognostic factor associated with breast cancer-specific survival (BCSS) and overall survival (OS). Those aged 50-64 years had significantly inferior BCSS (P<0.001) and OS (P<0.001) than those aged <35 years. Patients aged ≥65 years also had significantly lower BCSS (P<0.001) and OS (P<0.001) than those aged <35 years. However, similar outcomes were found between those aged 35-49 and <35 years.

**Conclusion:**

Our study suggests that different age groups may affect the metastatic patterns among patients with dnMBC and the survival of younger patients is more favorable than those of older patients.

## Introduction

Breast cancer (BC) is the most common type of malignant neoplasm in women (1). Due to the initiation of BC screening, approximately 65% of patients were diagnosed with early-stage BC and the 5-year overall survival (OS) has exceeded 90% in this population (2, 3). However, 5% of patients still present with distant metastasis (DM) disease at BC diagnosis (*de novo* metastatic breast cancer, dnMBC). Bone metastasis was the most common site of DM in BC, followed by lung, liver, and brain (4). With a deep understanding of BC, it is found that BC is a highly heterogeneous disease (5). According to the specific biological characteristics of different BC subtypes (BCS), corresponding therapeutic strategies such as radiotherapy, chemotherapy, targeted therapy, endocrine therapy, or immunotherapy have been developed in recent decades (6). According to the data from the United States (US) between 2012 and 2018, patients with dnMBC had the lowest 5-year OS rate (29%), which was much lower than stage I (>99%), stage II (93%), and stage III (75%) (7). A study from the US found that the 5-year disease-specific survival rate of the dnMBC increased from 28% to 55% in those diagnosed in 1990 and 2010, respectively (8). However, a cohort study from Germany found that the survival of this population hardly changed between 1978 and 2013 (9). Identifying clinical risk factors closely correlated with DM can offer insights into the underlying mechanism of advanced BC and inform the development of treatment strategies.

BC is an age-related disease. Age, as a prominent risk factor of tumorigenesis in BC, contributes greatly to the development of metastasis, possibly due to age-related changes in patient homeostasis and the tumor microenvironment (10, 11). The median age of those with non-metastatic patients was 62 years (12). Disproportionately higher rates of BC-related death have been found in those with younger BC as well as elderly BC (13–15). However, the age thresholds were inconsistent in the above studies. The median age in those with dnMBC was 61 years, which was similar to those with non-metastatic patients (16, 17). Young BC patients typically display more aggressive tumor characteristics, whereas older patients often experience a poorer prognosis (18, 19). However, the extent to which age is closely associated with metastasis in BC patients remains largely uncertain. Is dnMBC the same disease in the elderly as in younger BC? In light of this, our study aimed to investigate the patterns of DM and survival outcomes among the age groups in this population.

## Materials and methods

### Patient selection

**Figure 1:**
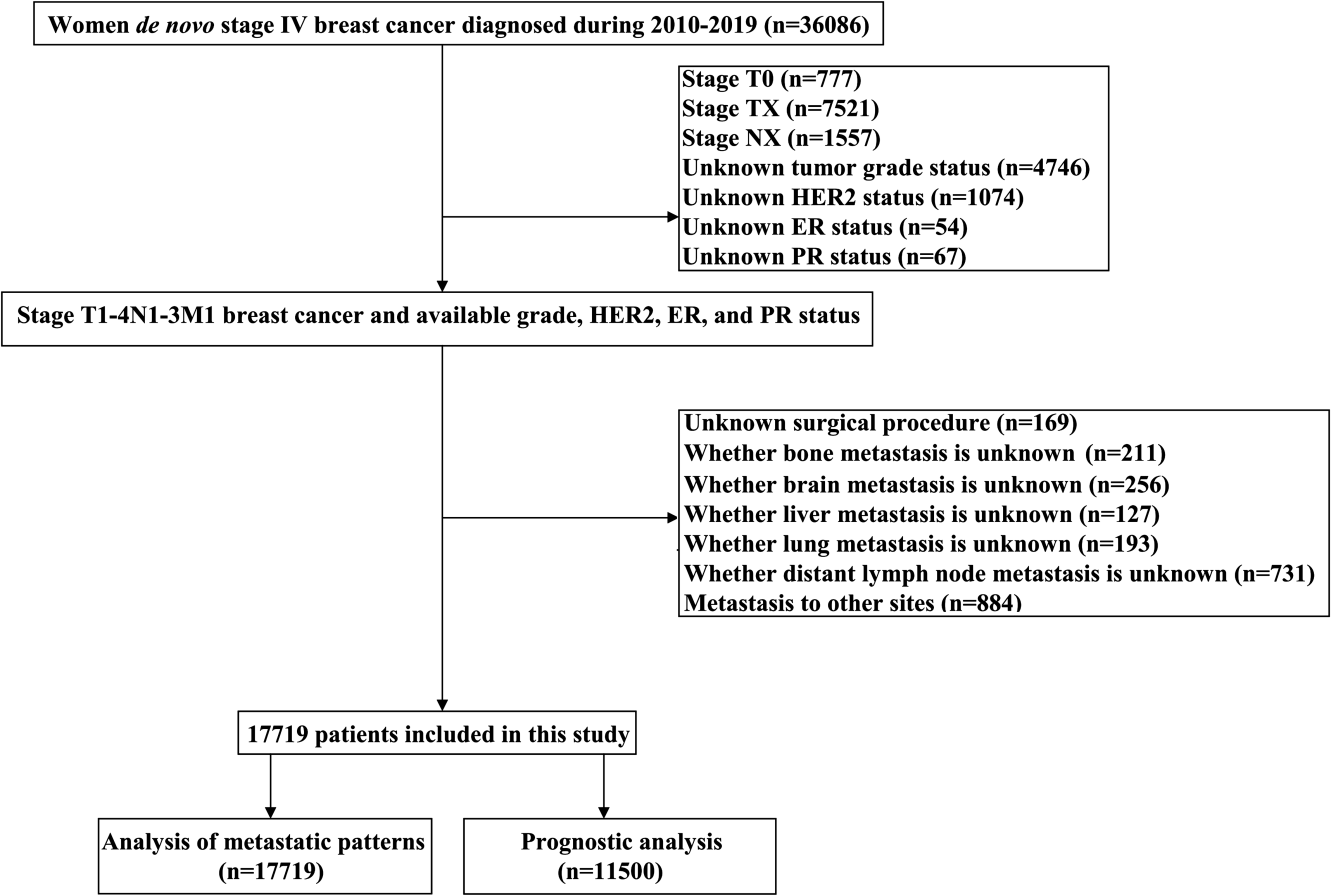
The patient selection flowchart.

The study data was retrieved from the Surveillance Epidemiology and End Results (SEER) database from 2010 to 2019 which was released in April 2022 (available at: https://seer.cancer.gov/), using the SEER*Stat software (version 8.3.9) (20). The SEER is a population-based dataset that covers approximately 30% population of the US, including demographic, clinicopathologic, diagnostic, first course of treatment, and survival information. The diagnosis of BC was identified using the International Classification of Disease for Oncology, Third Edition, which were all pathologically confirmed. The following inclusion criteria were used: 1) female with dnMBC; 2) available sites of DM included bone, lung, liver, brain, or distant lymph nodes; 3) available information included tumor grade, estrogen receptor (ER), progesterone receptor (PR), and human epidermal growth factor receptor 2 (HER2) status. The patient selection flowchart in this study has listed in **Figure 1**. Patients with an unknown tumor (T) stage, unknown nodal (N) stage, or unknown surgical procedure were excluded. This study did not require approval from the institutional review board due to the de-identified information in the SEER program.

### Measures

The following patient and tumor characteristics were included: age at diagnosis, race, histological subtype, grade, T stage, N stage, hormone receptor (HoR) status, HER2 status, BCS, and treatment. All the patients were divided into four age groups: <35 years, 35-49 years, 50-64 years, and ≥65 years, these cut-offs having been selected based on previously reported studies (21–24). BCS was classified into four subtypes: HoR+/HER2-, HoR+/HER2+, HoR-/HER2+, and HoR-/HER2-. The main endpoints of our study were breast cancer-specific survival (BCSS) and OS. BCSS was calculated as the time from the initial diagnosis of BC to the date of BC-specific death or last follow-up. OS was defined as the time from the initial diagnosis of BC to the date of death due to any cause. We used the variable names COD_ to_ site_record in the SEER database to analyze the cause of death in patients. This record was introduced to account for several newly valid International Classification of Diseases-10 codes and includes both cancer and non-cancer causes of death.

### Statistical analysis

The patient and tumor characteristics were compared using the Chi-square test. Multivariate logistic regression analyses were used to determine the relationship between different predictive factors and the metastatic patterns. The BCSS and OS curves were described using the Kaplan-Meier methods and compared by the log-rank test. A Cox proportional hazard regression model was based on the assumption that hazard rates were proportional over time. Variables with a P value <0.1 in the univariate Cox regression model were incorporated into the multivariate Cox proportional analysis to determine the prognostic factors that were significantly related to the survival outcomes. For the multivariable analyses, we used time-dependent variable approaches to check the proportional hazards (PH) assumption. Statistical analyses were conducted by the IBM SPSS 22.0 package (IBM Corp., Armonk, NY, USA) and a two-sided P-value <0.05 was considered statistically significant.

## Results

### Patient demographics

We included 17719 patients in this study. The patient characteristics have listed in **Table 1**. The median age was 61 years (range, 15-99 years), and 3.6% (n=638), 18.6% (n=3290), 38.0% (n=6725), and 39.9% (n=7066) were aged <35, 35-49, 50-64, and ≥65 years, respectively. There were 74.4% (n=13198) of patients were white, 77.8% (n=13777) were invasive ductal carcinoma subtype, 51.7% (n=9166) were stage T3-4 disease, and 66.9% (n=13619) were nodal positive diseases. Regarding BCS, 60.1% (n=10646), 16.8% (n=2976), 9.0% (n=1588), and 14.2% (n=2509) of patients had HoR+/HER2-, HoR+/HER2+, HoR-/HER2+, and HoR-/HER2- subtypes, respectively. Those with older age were more likely to be white race, invasive lobular carcinoma subtype, T4 diseases, ER+ diseases, and HER2- diseases (all P<0.001). However, those with older age were less likely to have N3 disease and poorly/undifferentiated disease (all P<0.001). Regarding treatment, patients of older age were less likely to receive surgery, chemotherapy as well as radiotherapy (all P<0.001). In those receiving surgical treatment for the primary breast tumors (n=6120), there were 1701 (27.8%) patients treated with breast-conserving surgery and 4419 (72.2%) treated with mastectomy. Patients with older age were more likely to receive breast-conserving surgery compared to those with younger age (P<0.001).

**Table 1:** Patient baseline characteristics according to age groups (n=17719).

Variables	n	<35 years (%)	35-49 years (%)	50-64 years (%)	≥65 years (%)	P
Race						
White	13198	414 (64.9)	2257 (68.6)	4842 (72.0)	5685 (80.5)	<0.001
Black	2843	153 (24.0)	615 (18.7)	1197 (17.8)	878 (12.4)	
Other	1678	71 (11.1)	418 (12.7)	686 (10.2)	506 (7.1)	
Histology						
IDC	13777	568 (89.0)	2676 (81.3)	5238 (77.9)	5295 (74.9)	<0.001
ILC	1705	12 (1.9)	214 (6.5)	626 (9.3)	853 (12.1)	
Other	2237	58 (9.1)	400 (12.2)	861 (12.8)	918 (13.0)	
Tumor stage						
T1	2448	73 (11.4)	428 (13.0)	914 (13.6)	1033 (14.6)	<0.001
T2	6105	215 (33.7)	1222 (37.1)	2202 (32.7)	2466 (34.9)	
T3	3247	175 (27.4)	681 (20.7)	1241 (18.5)	1150 (16.3)	
T4	5919	175 (27.4)	959 (29.1)	236 (35.2)	2417 (34.2)	
Nodal stage						
N0	4100	103 (16.1)	588 (17.9)	1386 (20.6)	2023 (28.6)	<0.001
N1	8368	324 (50.8)	1684 (51.2)	3155 (46.9)	3205 (45.4)	
N2	2168	77 (12.1)	407 (12.4)	904 (13.4)	780 (11.0)	
N3	3083	134 (21.0)	611 (18.6)	1280 (19.0)	1058 (15.0)	
Tumor grade						
G1	1452	20 (3.1)	208 (6.3)	489 (7.3)	735 (10.4)	<0.001
G2	7648	204 (32.0)	1325 (40.3)	2815 (41.9)	3304 (46.8)	
G3	8619	414 (64.9)	1757 (53.4)	3421 (50.9)	3027 (42.8)	
ER status						
Negative	4355	208 (32.6)	847 (25.7)	1752 (26.1)	1548 (21.9)	<0.001
Positive	13364	430 (67.4)	2443 (74.3)	4973 (73.9)	5518 (78.1)	
PR status						
Negative	6812	274 (42.9)	1186 (36.0)	2798 (41.6)	2554 (36.1)	<0.001
Positive	10907	364 (57.1)	2104 (64.0)	3927 (58.4)	4512 (63.9)	
HER2 status						
Negative	13155	382 (59.9)	2293 (69.7)	4842 (72.0)	5638 (79.8)	<0.001
Positive	4564	256 (40.1)	997 (30.3)	1883 (28.0)	1428 (20.2)	
Surgery						
No	11599	346 (54.2)	1932 (58.7)	4332 (64.4)	4989 (70.6)	<0.001
Yes	6120	292 (45.8)	1358 (41.3)	2393 (35.6)	2077 (29.4)	
Chemotherapy						
No	6219	70 (11.0)	662 (20.1)	1854 (27.6)	3633 (51.4)	<0.001
Yes	11500	568 (89.0)	2628 (79.9)	4871 (72.4)	3433 (48.6)	
Radiotherapy						
No	11474	354 (55.5)	1912 (58.1)	4179 (62.1)	5029 (71.2)	<0.001
Yes	5835	262 (41.1)	272 (38.7)	2379 (35.4)	1922 (27.2)	
Unknown	410	22 (3.4)	106 (3.2)	167 (2.5)	115 (1.6)	

IDC, invasive ductal carcinoma; ILC, invasive lobular carcinoma; T, tumor; N, nodal; G1, well differentiated; G2, moderately differentiated; G3, poorly/undifferentiated; ER, estrogen receptor; PR, progesterone receptor; HER2, human epidermal growth factor receptor 2.

### Metastasis patterns

**Figure 2:**
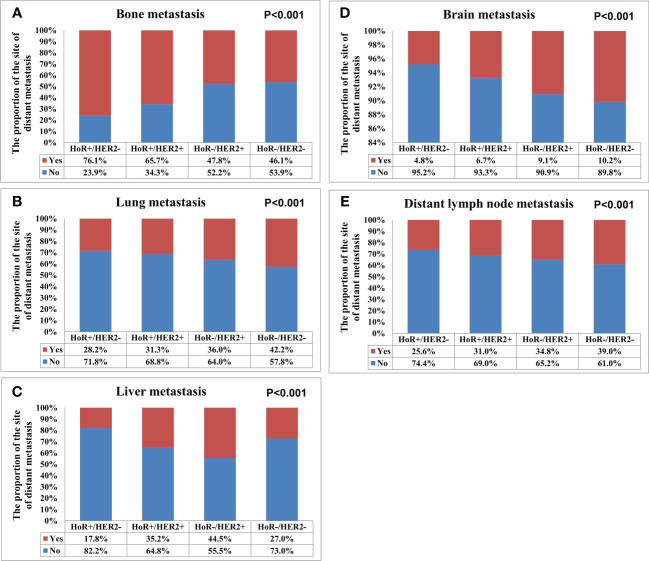
The patterns of distant metastases by different breast cancer subtypes in *de novo* metastatic breast cancer patients (**A**, bone metastasis; **B**, lung metastasis; **C**, liver metastasis; **D**, brain metastasis; **E**, distant lymph node metastasis).

**Figure 3:**
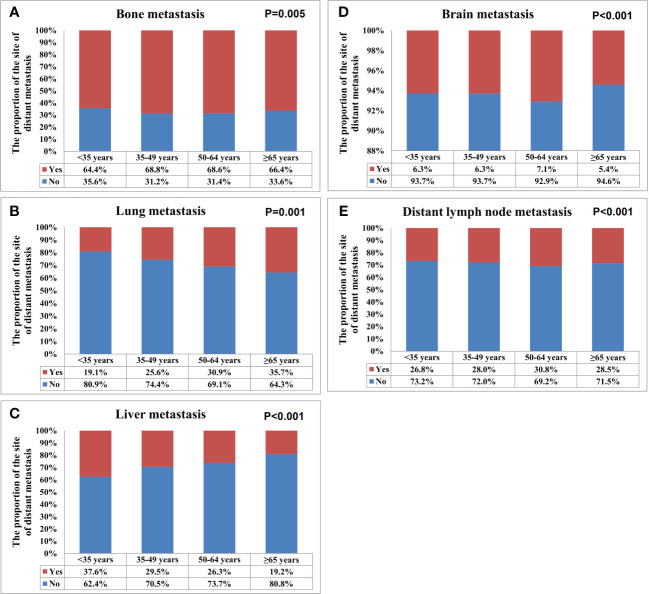
The patterns of distant metastases by different age groups in *de novo* metastatic breast cancer patients (**A**, bone metastasis; **B**, lung metastasis; **C**, liver metastasis; **D**, brain metastasis; **E**, distant lymph node metastasis).

A total of 28155 metastatic sites were identified in this study (**Table 2**). The SEER database only records distant organ metastases and does not include information on the number of metastatic lesions within specific metastatic organs. Therefore, the specific metastatic sites indicated the specific metastatic organs in this study. Bone was the most common metastatic site (n=11977, 42.5%), followed by lung (n=5566, 19.8%), distant lymph nodes (n=5176, 18.4%), liver (n=4331, 15.4%), and brain (n=1105, 3.9%). There were 10683 (60.3%), 4385 (24.7%), 1986 (11.2%), 581 (3.3%), and 84 (0.5%) patients who had one, two, three, four, and five metastatic sites, respectively. Patients with HoR+/HER2- were more likely to have bone metastasis (76.1% vs. 46.1-65.7%, P<0.001), those with HoR+/HER2+ and HoR-/HER2+ subtypes were more likely to have liver metastasis (35.2-44.5% vs. 17.8-27.0%, P<0.001), and those with HoR-/HER2- disease were more likely to have brain metastasis (10.2% vs. 4.8-9.1%, P<0.001), lung metastasis (42.2% vs. 28.2-36.0%, P<0.001), and distant lymph nodes metastasis (39.0% vs. 25.6-34.8%, P<0.001) (**Figure 2**). There was a small difference in the proportion of bone (**Figure 3A**), brain (**Figure 3D**), and distant lymph node metastasis (**Figure 3E**) among the four age subgroups. However, the risk of lung metastasis was significantly higher and the risk of liver metastasis was significantly lower in the older patients. There were 19.1%, 25.6%, 30.9%, and 35.7% of patients with lung metastasis in those aged <35, 35-49, 50-64, and ≥65 years, respectively (**Figure 3B**). Moreover, 37.6%, 29.5%, 26.3%, and 19.2% of patients had liver metastasis in those aged <35, 35-49, 50-64, and ≥65 years, respectively (**Figure 3C**). We found similar distributions of the sites of DM among the four age subgroups in patients with single-site metastasis. Moreover, similar distributions of the sites of DM among the four age subgroups were found after stratification by the BCS.

**Table 2:** The patterns of distant metastasis in *de novo* metastatic breast cancer patients (n=28155).

Number of metastatic sites	Patterns of distant metastasis	N	%
Single metastatic site (n=10683)	Bone	6318	59.1
	Distant lymph nodes	1589	14.9
	Lung	1486	13.9
	Liver	1150	10.8
	Brain	140	1.3
Second metastatic sites (n=4385)	Bone+lung	1090	24.9
	Bone+liver	1074	24.5
	Bone+distant lymph nodes	890	20.3
	Lung+distant lymph nodes	552	12.6
	Liver+lung	260	5.9
	Bone+brain	208	4.7
	Liver+distant lymph nodes	175	4.0
	Brain+lung	82	1.9
	Brainl+distant lymph nodes	35	0.8
	Brain+liver	19	0.4
Three metastatic sites (n=1986)	Bone+lung+distant lymph nodes	710	35.8
	Bone+lung+liver	438	22.1
	Bong+liver+distant lymph nodes	374	18.8
	Liver+lung+distant lymph nodes	156	7.9
	Bone+brain+lung	98	4.9
	Bone+brain+liver	68	3.4
	Bong+brain+distant lymph nodes	60	3.0
	Brain+lung+distant lymph nodes	45	2.3
	Brain+liver+lung	24	1.2
	Brain+liver+distant lymph nodes	13	0.7
Four metastatic sites (n=581)	Bone+lung+liver+distant lymph nodes	352	60.6
	Bone+brain+liver+lung	88	15.1
	Bone+brain+lung+distant lymph nodes	85	14.6
	Bone+brain+liver+distant lymph nodes	40	6.9
	Brain+liver+lung+distant lymph nodes	16	2.8
Five metastatic sites (n=84)	Bone+brain+lung+liver+distant lymph nodes	84	100

### The association between age groups and metastatic patterns

The multivariate logistic regression analysis was conducted to analyze the association between the age groups and the metastatic patterns (**Table 3**). The following variables were included in the multivariate logistic regression model: specific site of DM, race, age, histology, tumor stage, nodal stage, ER status, PR status as well as HER2 status. The results indicated that age was the independent risk factor for lung and liver metastases. The risk of lung metastasis increased with age, those aged 35-49, 50-64, and ≥65 years had 1.631 (P<0.001), 2.177 (P<0.001), and 2.963 (P<0.001) times of lung metastasis compared to those aged <35 years. Moreover, the risk of liver metastasis decreased with age, those aged 35-49, 50-64, and ≥65 years had 0.786 (P<0.001), 0.677 (P<0.001), and 0.494 (P<0.001) time of liver metastasis compared to those aged <35 years. Age was also the independent predictive factor for bone and distant lymph node metastases, but there was no significant change with age. Age was not an independent risk factor for brain metastasis.

**Table 3:** Multivariate logistic regression analysis of the association between age at diagnosis and the patterns of distant metastasis in *de novo* metastatic breast cancer patients.

Variables	Years	OR	95%CI	P
Bone metastasis	<35	1		
	35-49	1.027	0.852-1.237	0.781
	50-64	0.996	0.833-1.191	0.966
	≥65	0.763	0.638-0.913	0.003
Lung metastasis	<35	1		
	35-49	1.631	1.314-2.023	<0.001
	50-64	2.177	1.769-2.679	<0.001
	≥65	2.963	2.408-3.647	<0.001
Liver metastasis	<35	1		
	35-49	0.786	0.655-0.943	0.010
	50-64	0.677	0.568-0.806	<0.001
	≥65	0.494	0.414-0.589	<0.001
Brain metastasis	<35	1		
	35-49	1.071	0.754-1.523	0.702
	50-64	1.188	0.849-1.663	0.314
	≥65	0.920	0.655-1.293	0.631
Distant lymph node metastasis	<35	1		
	35-49	1.171	0.960-1.428	0.118
	50-64	1.359	1.124-1.644	0.002
	≥65	1.393	1.150-1.685	0.001

CI, confidence interval; OR, odds ratio.

### Prognostic analysis

**Figure 4:**
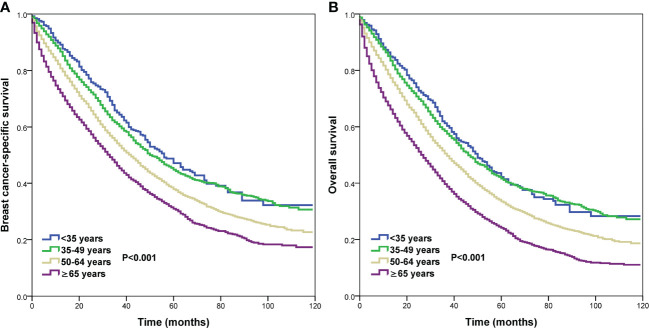
Comparison of survival outcomes by different age groups in *de novo* metastatic breast cancer patients (**A**, breast cancer-specific survival; **B**, overall survival).

We conducted the prognostic analysis on patients with HoR+/HER- and patients who received chemotherapy in the HoR+/HER2+, HoR−/HER2+, and HoR−/HER2− subtypes (n=16218). All variables in the univariate Cox regression model were P value <0.1 and were incorporated into the multivariate Cox proportional analysis. In addition, all variables in the multivariate Cox regression analyses satisfied the PH hypothesis with its hazard ratio [HR] value changes with time. The results of the multivariate Cox regression analyses indicated that age was the independent prognostic factor related to survival outcomes (**Table 3**, **Supplementary Table 1**). Those aged 50-64 years had significantly lower BCSS (HR 1.258, P<0.001) and OS (HR 1.227, P<0.001) compared to those aged <35 years. Patients aged ≥65 years also had significantly lower BCSS (HR 1.648, P<0.001) and OS (HR 1.722, P<0.001) compared to those aged <35 years. However, similar BCSS (P=0.262) and OS (P=0.681) were found between those aged 35-49 and <35 years. The survival curves of different age groups have shown in **Figure 4**. Patients with bone (BCSS, HR 1.290, P<0.001; OS, HR 1.238, P<0.001), lung (BCSS, HR 1.164, P<0.001; OS, HR 1.156, P<0.001), liver (BCSS, HR 1.580, P<0.001; OS, HR 1.517, P<0.001), and brain metastasis (BCSS, HR 1.882, P<0.001; OS, HR 1.881, P<0.001), and ≥2 metastatic sites (BCSS, HR 1.180, P<0.001; OS, HR 1.155, P<0.001) also had inferior BCSS and OS, while those with distant lymph node metastasis (BCSS, HR 0.994, P=0.863; OS, HR 1.104, P=0.648) had no significant effect on survival outcomes (**Supplementary Table 1**).

We conducted sensitivity analyses to determine the effect of age on survival after stratification by the metastatic sites (**Table 4**). The following variables were included in the multivariate Cox regression models: race, age, histology, tumor stage, nodal stage, ER status, PR status, HER2 status, surgery, radiotherapy as well as the specific site of DM. The results also showed that age was the independent prognostic factor for BCSS and OS regardless of the sites of DM.

**Table 4:** Multivariate Cox regression analyses of the association between age at diagnosis and the breast cancer-specific survival and overall survival in *de novo* metastatic breast cancer patients (n=16218).

Variables	Years	BCSS			OS		
		HR	95%CI	P	HR	95%CI	P
Entire cohort	<35	1			1		
	35-49	1.081	0.944-1.238	0.262	1.027	0.904-1.167	0.681
	50-64	1.258	1.104-1.433	<0.001	1.227	1.086-1.387	<0.001
	≥65	1.648	1.444-1.880	<0.001	1.722	1.523-1.948	<0.001
Bone metastasis	<35	1			1		
	35-49	1.041	0.883-1.227	0.631	1.007	0.862-1.177	0.930
	50-64	1.242	1.061-1.455	0.007	1.862	1.059-1.426	0.007
	≥65	1.652	1.408-1.937	<0.001	1.484	1.484-2.004	<0.001
Lung metastasis	<35	1			1		
	35-49	0.923	0.709-1.201	0.551	0.896	0.698-1.105	0.392
	50-64	1.035	0.803-1.333	0.792	1.043	0.820-1.326	0.732
	≥65	1.309	1.015-1.689	0.038	1.407	1.105-1.790	0.006
Liver metastasis	<35	1			1		
	35-49	1.132	0.912-1.405	0.263	1.095	0.892-1.345	0.384
	50-64	1.398	1.137-1.719	0.001	1.361	1.119-1.656	0.002
	≥65	1.957	1.581-2.422	<0.001	1.986	1.624-2.430	<0.001
Brain metastasis	<35	1			1		
	35-49	0.983	0.629-1.535	0.938	1.054	0.677-1.640	0.817
	50-64	1.117	0.729-1.713	0.611	1.246	0.816-1.905	0.309
	≥65	1.672	1.074-2.602	0.023	1.881	1.216-2.909	0.005
Distant lymph node metastasis	<35	1			1		
	35-49	1.186	0.921-1.528	0.185	1.162	0.917-1.473	0.215
	50-64	1.357	1.066-1.727	0.013	1.309	1.044-1.642	0.020
	≥65	1.540	1.204-1.968	<0.001	1.602	1.273-2.015	<0.001

CI, confidence interval; HR, hazard ratio; BCSS, breast cancer-specific survival; OS, overall survival.

**Figure 5:**
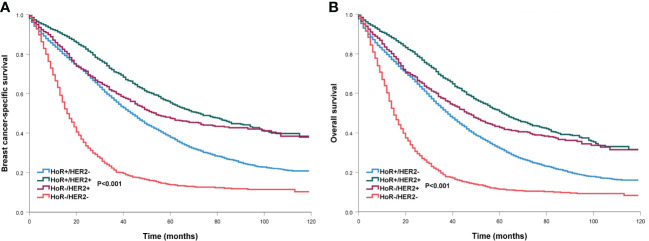
Comparison of survival outcomes by different breast cancer subtypes in *de novo* metastatic breast cancer patients (**A**, breast cancer-specific survival; **B**, overall survival).

When stratified by the sites of DM (**Table 5**), HoR-/HER2- subtype was consistently associated with an inferior BCSS (HR for any site: 2.469, 95% CI 2.318-2.630; bone: 2.870, 95% CI 2.634-3.126; lung: 2.109, 95% CI 1.902-2.339; liver: 2.092, 95% CI 1.854-2.360; brain: 2.055, 95%CI 1.669-2.530; distant lymph node: 2.339, 95%CI 2.102-2.602) and OS (HR for any site: 2.329, 95% CI 2.190-2.471; bone: 2.720, 95% CI 2.508-2.951; lung: 1.969, 95% CI 1.786-2.172; liver: 2.020, 95% CI 1.779-2.267; brain: 1.795, 95%CI 1.469-2.194; distant lymph node: 2.202, 95%CI 1.989-2.437) regardless of DM site. The BCSS and OS curves among the four subtypes have listed in **Figure 5**. Those with HoR+/HER2+ disease had better BCSS and OS compared to those with HoR+/HER2- disease regardless of DM site. However, comparable BCSS and OS were found between HoR+/HER2- and HoR-/HER2+ subtypes in those with bone, lung, brain, and distant lymph node metastases.

**Table 5:** Multivariate Cox regression analyses of the association between breast cancer subtypes and the breast cancer-specific survival and overall survival according to the site of distant metastasis in *de novo* metastatic breast cancer patients (n=16218).

Variables	Subtypes	BCSS			OS		
		HR	95%CI	P	HR	95%CI	P
Entire cohort	HoR+/HER2-	1			1		
	HoR+/HER2+	0.632	0.586-0.681	<0.001	0.640	0.597-0.684	<0.001
	HoR-/HER2+	0.823	0.752-0.900	<0.001	0.829	0.762-0.902	<0.001
	HoR-/HER2-	2.469	2.318-2.630	<0.001	2.326	2.190-2.471	<0.001
Bone metastasis	HoR+/HER2-	1			1		
	HoR+/HER2+	0.654	0.598-0.716	<0.001	0.655	0.602-0.713	<0.001
	HoR-/HER2+	0.887	0.784-1.004	0.057	0.898	0.800-1.007	0.067
	HoR-/HER2-	2.870	2.634-3.126	<0.001	2.720	2.508-2.951	<0.001
Lung metastasis	HoR+/HER2-	1			1		
	HoR+/HER2+	0.665	0.586-0.754	<0.001	0.658	0.585-0.741	<0.001
	HoR-/HER2+	0.913	0.790-1.055	0.216	0.914	0.799-1.046	0.190
	HoR-/HER2-	2.109	1.902-2.339	<0.001	1.969	1.786-2.172	<0.001
Liver metastasis	HoR+/HER2-	1			1		
	HoR+/HER2+	0.552	0.489-0.624	<0.001	0.566	0.504-0.635	<0.001
	HoR-/HER2+	0.646	0.564-0.740	<0.001	0.640	0.562-0.728	<0.001
	HoR-/HER2-	2.092	1.854-2.360	<0.001	2.020	1.779-2.267	<0.001
Brain metastasis	HoR+/HER2-	1			1		
	HoR+/HER2+	0.712	0.557-0.911	0.007	0.707	0.562-0.891	0.003
	HoR-/HER2+	1.271	0.981-1.647	0.069	1.179	0.920-1.512	0.193
	HoR-/HER2-	2.055	1.669-2.530	<0.001	1.795	1.469-2.194	<0.001
Distant lymph node metastasis	HoR+/HER2-	1			1		
	HoR+/HER2+	0.648	0.567-0.740	<0.001	0.654	0.578-0.740	<0.001
	HoR-/HER2+	0.924	0.796-1.072	0.297	0.905	0.787-1.041	0.164
	HoR-/HER2-	2.339	2.102-2.602	<0.001	2.202	1.989-2.437	<0.001

CI, confidence interval; HR, hazard ratio; BCSS, breast cancer-specific survival; OS, overall survival; HoR, hormone receptor; HER2, human epidermal growth factor receptor 2.

When stratified by age groups (**Table 6**), the HoR-/HER2- subtype was consistently associated with an inferior BCSS (HR for aged <35 years: 2.584, 95% CI 1.828-3.651; 35-49 years: 3.415, 95% CI 2.942-3.964; 50-64 years: 2.373, 95% CI 2.143-2.627; ≥65 years: 2.188, 95% CI 1.956-2.446) and OS (HR for aged <35 years: 2.539, 95% CI 1.832-3.521; 35-49 years: 3.284, 95% CI 2.846-3.791; 50-64 years: 2.291, 95% CI 2.080-2.524; ≥65 years: 1.925, 95% CI 1.733-2.139) regardless of age groups. In addition, those with HoR+/HER2+ and HoR-/HER2+ subtypes had significantly better BCSS and OS compared to HoR+/HER2- subtype regardless of age groups.

**Table 6:** Multivariate Cox regression analyses of the association between breast cancer subtypes and the breast cancer-specific survival and overall survival according to the age groups in *de novo* metastatic breast cancer patients.

Age (years)	Subtypes	BCSS			OS		
		HR	95%CI	P	HR	95%CI	P
<35	HoR+/HER2-	1			1		
	HoR+/HER2+	0.385	0.267-0.555	<0.001	0.449	0.321-0.628	<0.001
	HoR-/HER2+	0.482	0.318-0.729	<0.001	0.565	0.387-0.824	0.003
	HoR-/HER2-	2.584	1.828-3.651	<0.001	2.539	1.832-3.521	<0.001
35-49	HoR+/HER2-	1			1		
	HoR+/HER2+	0.463	0.389-0.552	<0.001	0.461	0.390-0.545	<0.001
	HoR-/HER2+	0.752	0.611-0.926	0.007	0.774	0.636-0.942	0.010
	HoR-/HER2-	3.415	2.942-3.964	<0.001	3.284	2.846-3.791	<0.001
50-64	HoR+/HER2-	1			1		
	HoR+/HER2+	0.585	0.521-0.656	<0.001	0.597	0.536-0.665	<0.001
	HoR-/HER2+	0.715	0.623-0.820	<0.001	0.712	0.625-0.811	<0.001
	HoR-/HER2-	2.373	2.143-2.627	<0.001	2.291	2.080-2.524	<0.001
≥65	HoR+/HER2-	1			1		
	HoR+/HER2+	0.631	0.549-0.724	<0.001	0.632	0.558-0.716	<0.001
	HoR-/HER2+	0.840	0.709-0.996	0.044	0.827	0.709-0.964	0.015
	HoR-/HER2-	2.188	1.956-2.446	<0.001	1.925	1.733-2.139	<0.001

CI, confidence interval; HR, hazard ratio; BCSS, breast cancer-specific survival; OS, overall survival; HoR, hormone receptor; HER2, human epidermal growth factor receptor 2.

## Discussion

In the present study, we investigated the effect of age on metastatic patterns and survival outcomes in dnMBC. Our results showed that there were certain specificities in dissemination to different distant organs by different age groups. In addition, patients of older age were associated with inferior prognoses in this population.

In our previous SEER study included 7575 patients diagnosed between 2010 and 2013, a total of 11140 sites of DM were found and bone (51.2%) was the most common metastatic site, followed by lung (23.2%), liver (20.1%), and brain (5.5%) (4). However, the distant lymph nodes were not analyzed in the study. In this study, we included 17719 patients with 28155 sites of DM, we found that bone was also the most common metastatic site (42.5%), followed by lung (19.8%), distant lymph nodes (18.4%), liver (15.4%), and brain (3.9%). Another study from Malmgren et al. also found that bone metastasis was the most common metastatic site (58%), followed by distant lymph nodes (33%), lung (21%), liver (21%), and brain (1%) (8). However, we should note that in the previous studies, 63.9-68% of patients had a single site of DM, and 60.3% were in our study. This suggests that the early diagnosis of advanced BC may not be further improved under the contemporary screening modes. The number of sites of DM was associated with survival outcomes in our study. Therefore, it is still necessary to explore strategies that can detect the disease in time.

There were specific metastatic patterns of different malignant tumors. In small-cell lung cancer, the liver was the most common metastatic site (44.8%), followed by bone (35.0%), brain (25.8%), and lung (20.6%) (25). In those with stage IV esophageal cancer, the lung was the most common metastatic site, followed by liver, bone, and brain (26). BC is a highly heterogeneous tumor (5). In our previous study, we found that patients with different BCS have various metastatic patterns. Patients with HoR+/HER2- and HoR+/HER2+ subtypes were more prone to bone metastases. Lung and brain metastases were common in HoR-/HER2+ and HoR-/HER2- subtypes and patients with HoR+/HER2+ and HoR-/HER2+ subtypes were more likely to have liver metastases (4). Similar results were found in the present study using a larger cohort. Therefore, patients with different tumor locations should have different priorities in surveillance, and individualized follow-up strategies should be formulated for different malignant tumors.

In this study, we further analyzed the association among the age groups and the metastatic patterns. We found that age could also affect the patterns of DM in BC, and these metastatic patterns by age groups were not affected by the BCS. There was a small difference in the proportion of bone, brain, and distant lymph node metastases among the four age groups. However, the risk of lung metastasis was higher and liver metastasis was lower in the older patients. A previous SEER study from Chen et al. included 4932 patients, they found that patients with older age had significantly higher rates of lung metastasis and a lower rate of distant lymph node metastasis. However, no significant differences in bone, brain, and liver metastasis were found among the age groups (27). We should notice that there were 5.0%, 63.7%, and 31.2% of patients were aged <50 years, 50-69 years, and ≥70 years in the above study. However, there were 22.2% of patients were aged <50 years in our study. A recent SEER study, which included 24155 patients diagnosed with dnMBC, categorized the age of these patients into four groups: ≤40 years, 41-60 years, 61-80 years, and >80 years (28). However, this analysis did not account for distant lymph node metastasis, limiting the ability to fully assess the patterns of DM within this cohort. Furthermore, the study observed a similar distribution of lung metastasis among the age groups of 61-80 and >80 years (32.4% vs. 33.6%, respectively). The age distribution of patients in our study is more highly represented in the literature (22–24, 29), providing a more comprehensive perspective. Therefore, the distribution of different age groups may affect the assessment of the metastatic patterns. However, Purushotham et al. found a decreased risk of bone and viscera metastases with the increase of age at diagnosis in those who developed DM during follow-up (30). Moreover, Hung et al. showed that younger patients were particularly prone to brain metastasis regardless of BCS (31).

The reasons behind the observed diverse patterns of DM among different age groups remain unclear. In this study, older patients were found to be more prone to lung metastases. This phenomenon may be attributed to chronic lung inflammation, such as chronic obstructive pulmonary disease, which is common in the elderly (32). Several studies have shown that potential mechanisms involving inflammatory cell neutrophils specifically support metastatic initiation and promote the awakening of dormant cancer cells in the lungs using mouse models (33, 34). Additionally, we observed a higher prevalence of liver metastases among younger patients. The increased risk of liver metastases in this demographic may be linked to a higher percentage of HER2+ disease in younger patients, consistent with previous studies including our own (4, 35). Given the high proportions of HER2+ subtypes in young patients, further research focusing on potential mechanisms of liver metastases in HER2+ BC may help in developing novel strategies for metastasis prevention. Moreover, it appears that tumor cell subsets interact differently with the tumor microenvironments in various distant metastatic organs, providing favorable conditions for the invasion and proliferation of tumor cells. We hypothesized that the immune system may also influence the metastatic patterns of patients. Younger patients tend to have stronger immune responses, resulting in differences in the tumor microenvironment. Therefore, it is essential to employ different strategies to treat different tumor cell subsets. Nonetheless, further investigation into the underlying molecular mechanisms is still needed, particularly concerning those with bone, brain, and distant lymph node metastases across different age groups.

In our study, older patients presented more often with larger tumor sizes. However, patients of older age were less likely to have N3 disease and have higher tumor grade. Therefore, older BC patients have special tumor biological behavior. The inferior outcomes in older patients might be related to lower administration of surgery, chemotherapy, radiotherapy, and endocrine therapy in older BC. Several studies also supported undertreatment in older BC (36, 37). Although most patients included in our multivariate survival analysis were treated with chemotherapy, the completion of chemotherapy, endocrine therapy, and targeted therapy were not recorded in the SEER database. Therefore, we could assume that elderly patients may have lower compliance with multimodal treatment. We also found that those who did not undergo surgery had worse survival. Although the results of the prospective studies did not find the survival benefit of additional surgery in this population (38, 39), many real-world studies including ours have found that surgical treatment could improve the survival of these patients (40, 41). Therefore, the local treatment strategy is still worth further exploration for this population, especially for the elderly. Moreover, some older women with BC may have biological invasive diseases, but their characteristics have not been fully determined (42, 43).

There were 19.8% of patients had lung metastasis in our study and the risk of lung metastasis increased with age. However, we found that age was not a prognostic factor affecting the survival of those with lung metastasis. The reason for this difference remains unclear. It should be noted that patients with HoR-/HER2- were more likely to have lung metastasis (42.2% vs. 28.2-36.0%, P<0.001) compared to other subtypes, which was similar to a previous study (44). Similarly, Ahn et al. found inferior survival for young patients with HoR+ disease, but not for those with HoR-/HER2- BC (45). In particular, 60-70% of metastatic patients who eventually died were diagnosed with lung metastasis (46). Therefore, the probability of HoR-/HER2- BC was higher among patients with lung metastasis, which may compensate for the differences in survival caused by age.

Only 3.6% of patients were diagnosed with dnMBC at <35 years of age in our study. However, BC in younger women is often diagnosed at more advanced stages of the disease. The main reason for this is the lack of screening, which is not recommended in this age group, as well as the longer delay in diagnosis. In addition, young women often have dense breast parenchyma, which could reduce the sensitivity and accuracy of digital mammography (47). There is no evidence of a mortality benefit from mammographic screening in women under the age of 35 years (48). Our results showed that younger patients were more likely to have N3 disease and poorly/undifferentiated disease. Therefore, screening is also needed for high-risk young women, and new screening methods should be explored. A previous study has shown worse 5-year survival in young women with non-metastatic BC at diagnosis compared with older, premenopausal patients (49). However, in our study, patients aged <35 years had comparable BCSS and OS compared to those with aged 35-49 years, while they had significantly better BCSS and OS than those aged ≥50 years. Moreover, those with HER2+ subtypes had significantly better BCSS and OS, while those with HoR-/HER2- had significantly inferior BCSS and OS in patients aged <35 years. Therefore, advances in systemic therapy have contributed to improving survival outcomes for young patients with dnMBC by tailoring treatments to their disease biology.

The median age at the time of diagnosis was 61 years, with approximately 40% of patients diagnosed at the age of 65 or older in our study. Despite the high prevalence of cancer in older patients, their participation in oncology clinical trials has traditionally been inadequate. Recent data indicated that only 1% of trials have enrolled solely patients aged 65 or 70 and older (50). In general, using upper age limits in clinical trials presents challenges due to the considerable heterogeneity of aging. This means that there is often no direct correlation between an older woman’s chronological age and her biological age. This is particularly significant for patients who may undergo chemotherapy or newer biological therapies, where published trials have typically included a few older patients, limiting information on both short-term and long-term toxicity among this demographic. This underrepresentation is often attributed to age bias among clinicians (51). However, studies have demonstrated that this healthy older age group tolerates treatment as well as younger patients, including breast and axillary surgery, chemotherapy, and radiotherapy. Therefore, it is important that older patients are actively recruited into studies evaluating novel agents and therapeutic approaches whenever possible and appropriate, as is recommended for all patients.

Our study had several limitations. First, miscoding and missing information inevitably existed in any retrospective studies. Second, there was no information concerning systemic treatment in different age groups, including the chemotherapy regimen, the completion of chemotherapy, endocrine therapy, targeted therapy, and immunotherapy. Third, although these are the common sites of DM in BC, metastases to other distant sites may influence the survival of BC patients. Fourth, information regarding patient comorbidities was inaccessible in the SEER database, and we could not adjust for patient comorbidities in the multivariable analysis. This limitation may have influenced estimations of survival and treatment selection. Moreover, older BC patients are frequently excluded from ongoing clinical trials, leading to inadequate treatment and inferior survival rates. Therefore, the correlation between age and BC mortality is far more complex, with potential confounding factors possibly contributing to better survival outcomes in younger women. Finally, only 3.6% of patients were under 35 years old in our study, which could limit the representativeness and generalizability of our findings. However, the incidence of dnMBC among young women has been steadily increasing over the last decades, while remaining stable in other age groups (52, 53).

## Conclusions

In conclusion, our study suggests that different age groups may affect the metastasis pattern of patients with dnMBC, and the survival of younger patients is relatively more favorable than those with older age. More studies are needed to fully verify the effect of age in predicting metastatic patterns and prognosis in this population.

## Data availability statement

The raw data supporting the conclusions of this article will be made available by the authors, without undue reservation.

## Ethics statement

The studies were conducted in accordance with the local legislation and institutional requirements. This study did not require approval from the institutional review board due to the deidentified information in the SEER program. Informed consent is not required because the data were extracted from the SEER database after obtaining the permission of the administrator. In addition, the privacy of the patients was well protected due to the anonymization and de-identification of the patient information.

## Author contributions

KL: Conceptualization, Data curation, Formal analysis, Visualization, Writing – original draft. A-LH: Conceptualization, Data curation, Formal analysis, Funding acquisition, Resources, Writing – original draft. X-QC: Conceptualization, Funding acquisition, Methodology, Resources, Supervision, Validation, Visualization, Writing – review & editing. S-GW: Conceptualization, Data curation, Formal analysis, Funding acquisition, Investigation, Methodology, Project administration, Resources, Software, Supervision, Validation, Visualization, Writing – review & editing.
