# Supplementary material for: Patterns of distant metastasis and survival outcomes in de novo metastatic breast cancer according to age groups
Source: Front Endocrinol (Lausanne). 2024 May 1;15:1385756. doi: 10.3389/fendo.2024.1385756 (PMC11094241; doi:10.3389/fendo.2024.1385756)
Supplement: Supplementary file 1 [file Table_1.doc]

**Supplementary Table 1.** The results of multivariate Cox regression analyses.

| Variables | BCSS | | | OS | | |
| --- | --- | --- | --- | --- | --- | --- |
|  | HR | 95%CI | P | HR | 95%CI | P |
| Age (years) |  |  |  |  |  |  |
| <35 | 1 |  |  | 1 |  |  |
| 35-49 | 1.081 | 0.944-1.239 | 0.258 | 1.028 | 0.905-1.167 | 0.676 |
| 50-64 | 1.258 | 1.105-1.433 | <0.001 | 1.228 | 1.087-1.387 | <0.001 |
| ≥65 | 1.647 | 1.444-1.8798 | <0.001 | 1.723 | 1.523-1.948 | <0.001 |
| Race |  |  |  |  |  |  |
| White | 1 |  |  | 1 |  |  |
| Black | 1.299 | 1.224-1.379 | <0.001 | 1.331 | 1.259-1.407 | <0.001 |
| Other | 0.925 | 0.851-1.174 | 0.069 | 0.927 | 0.857-1.002 | 0.927 |
| Histology |  |  |  |  |  |  |
| IDC | 1 |  |  | 1 |  |  |
| ILC | 1.258 | 1.162-1.362 | <0.001 | 1.236 | 1.148-1.330 | 0.005 |
| Other | 1.099 | 1.029-1.174 | 0.005 | 1.106 | 1.040-1.175 | 0.001 |
| Tumor stage |  |  |  |  |  |  |
| T1 | 1 |  |  | 1 |  |  |
| T2 | 1.085 | 1.005-1.171 | 0.036 | 1.102 | 1.027-1.183 | 0.007 |
| T3 | 1.207 | 1.109-1.314 | <0.001 | 1.200 | 1.109-1.299 | <0.001 |
| T4 | 1.397 | 1.293-1.510 | <0.001 | 1.411 | 1.313-1.517 | <0.001 |
| Nodal stage |  |  |  |  |  |  |
| N0 | 1 |  |  | 1 |  |  |
| N1 | 0.958 | 0.886-1.036 | 0.282 | 0.935 | 0.885-0.988 | 0.016 |
| N2 | 1.032 | 0.931-1.144 | 0.551 | 0.980 | 0.908-1.057 | 0.600 |
| N3 | 1.147 | 1.046-1.257 | 0.004 | 1.038 | 0.969-1.112 | 0.286 |
| Tumor grade |  |  |  |  |  |  |
| G1 | 1 |  |  | 1 |  |  |
| G2 | 1.276 | 1.162-1.401 | <0.001 | 1.214 | 1.116-1.320 | <0.001 |
| G3 | 1.796 | 1.630-1.978 | <0.001 | 1.646 | 1.508-1.797 | <0.001 |
| ER status |  |  |  |  |  |  |
| Negative | 1 |  |  | 1 |  |  |
| Positive | 0.693 | 0.645-0.744 | <0.001 | 0.707 | 0.661-0.756 | <0.001 |
| PR status |  |  |  |  |  |  |
| Negative | 1 |  |  | 1 |  |  |
| Positive | 0.670 | 0.630-0.713 | <0.001 | 0.698 | 0.659-0.739 | <0.001 |
| HER2 status |  |  |  |  |  |  |
| Negative | 1 |  |  | 1 |  |  |
| Positive | 0.397 | 0.372-0.423 | <0.001 | 0.420 | 0.396-0.446 | <0.001 |
| Surgery |  |  |  |  |  |  |
| No | 1 |  |  | 1 |  |  |
| Yes | 0.774 | 0.751-0.797 | <0.001 | 0.773 | 0.752-0.794 | <0.001 |
| Radiotherapy |  |  |  |  |  |  |
| No | 1 |  |  | 1 |  |  |
| Yes | 0.961 | 0.913-1.011 | 0.128 | 0.937 | 0.894-0.983 | 0.008 |
| Unknown | 0.955 | 0.694-1.036 | 0.780 | 0.905 | 0.668-1.227 | 0.521 |
| Bone metastasis |  |  |  |  |  |  |
| No | 1 |  |  | 1 |  |  |
| Yes | 1.290 | 1.214-1.370 | <0.001 | 1.238 | 1.170-1.309 | <0.001 |
| Lung metastasis |  |  |  |  |  |  |
| No | 1 |  |  | 1 |  |  |
| Yes | 1.164 | 1.095-1.238 | <0.001 | 1.156 | 1.091-1.224 | <0.001 |
| Liver metastasis |  |  |  |  |  |  |
| No | 1 |  |  | 1 |  |  |
| Yes | 1.580 | 1.486-1.680 | <0.001 | 1.517 | 1.432-1.607 | <0.001 |
| Brain metastasis |  |  |  |  |  |  |
| No | 1 |  |  | 1 |  |  |
| Yes | 1.882 | 1.720-2.059 | <0.001 | 1.881 | 1.728-2.047 | <0.001 |
| Distant lymph node metastasis |  |  |  |  |  |  |
| No | 1 |  |  | 1 |  |  |
| Yes | 0.994 | 0.933-1.060 | 0.863 | 1.104 | 0.955-1.077 | 0.648 |
| Number of metastatic sites |  |  |  |  |  |  |
| 1 | 1 |  |  | 1 |  |  |
| ≥2 | 1.180 | 1.101-1.264 | <0.001 | 1.155 | 1.083-1.231 | <0.001 |

IDC, invasive ductal carcinoma; ILC, invasive lobular carcinoma; T, tumor; N, nodal; G1, well differentiated; G2, moderately differentiated; G3, poorly/undifferentiated; ER, estrogen receptor; PR, progesterone receptor; HER2, human epidermal growth factor receptor 2; CI, confidence interval; HR, hazard ratio; BCSS, breast cancer-specific survival; OS, overall survival.
